# Paediatric antiretroviral therapy challenges with emerging integrase resistance

**DOI:** 10.1097/COH.0000000000000876

**Published:** 2024-07-05

**Authors:** Alasdair Bamford, Lisa Hamzah, Anna Turkova

**Affiliations:** a Great Ormond Street Hospital for Children NHS Foundation Trust; b UCL Great Ormond Street Institute of Child Health; c MRC Clinical Trials Unit at UCL; d St George's University Hospital NHS Trust, London, UK

**Keywords:** children, formulations, HIV, integrase inhibitors, resistance

## Abstract

**Purpose of review:**

Universal antiretroviral (ART) coverage and virological suppression are fundamental to ending AIDS in children by 2030. Availability of new paediatric dolutegravir (DTG)-based ART formulations is a major breakthrough and will undoubtedly help achieve this goal, but treatment challenges still remain.

**Recent findings:**

Paediatric formulations remain limited compared to those for adults, especially for young children, those unable to tolerate DTG or with DTG-based first-line ART failure. Tenofovir alafenamide is virologically superior to standard-of-care backbone drugs in second-line, but paediatric formulations are not widely available. The roles of resistance testing and recycling of backbone drugs following first-line ART failure remain to be determined. Results of trials of novel treatment strategies including dual therapy and long-acting agents are awaited. Although numbers are currently small, safe and effective ART options are urgently required for children developing DTG resistance.

**Summary:**

The antiretroviral treatment gap between adults and children persists. The potential benefits from rollout of new paediatric DTG-based fixed-dose combination ART for first-line treatment are considerable. However, children remain disadvantaged when DTG-based first-line ART fails or cannot be used. Research efforts to address this inequity require prioritisation in order to ensure health outcomes are optimised for all ages in all settings.

## INTRODUCTION

With recent advances in paediatric antiretroviral formulation development, including child-friendly fixed-dose combinations (FDCs), universal access to high potency, safe, cost-effective antiretroviral therapy (ART) appears achievable in theory. However, ART coverage for children remains much lower than that for adults, especially in low and middle-income countries (LMIC) [1,2]. A high proportion of children remain undiagnosed and of those on ART, rates of virological failure (VF) are higher than for adults [2]. Severe immunosuppression both at first presentation and also while receiving ART is still common [3]. Higher VF rates are in part attributable to current ART regimens that bring challenges to adherence, particularly for young children and those not on first-line treatment. It is hoped that the inequity will be addressed by rapid rollout of newer paediatric FDCs however barriers remain in providing the necessary range of ART options for treatment-naïve and experienced children.

Standard ART recommendations for children include an anchor drug from one of three classes [integrase inhibitor (INSTI), protease inhibitor (PI) or nonnucleoside reverse transcriptase inhibitor (NNRTI)], plus two nucleoside/nucleotide reverse transcriptase inhibitors (NRTIs). Current paediatric guidelines recommend second-generation INSTIs, predominately dolutegravir (DTG), as preferred first-line anchor drug for all above 4 weeks and 3 kg [4–6]. Robust evidence for this recommendation (including evidence for simplified dosing and dispersible tablets) has been provided by IMPAACT P1093, IMPAACT 2019 and ODYSSEY trials [7–14]. Observational data following transition to DTG-based therapy in multiple settings confirms real-world effectiveness [15^▪^,16^▪^,17^▪^]. DTG is also recommended for those experiencing VF on non-INSTI-based first-line therapy, supported by evidence from ODYSSEY [7,9] and CHAPAS-4 trials [18].

NRTI backbone recommendations are more complex, varying according to age and formulation availability. However, preferred first-line ART generally includes lamivudine (3TC) or emtricitabine (FTC) (interchangeable and therefore collectively referred to here as XTC) with either abacavir (ABC) for younger children or tenofovir (disoproxil fumarate (TDF) or alafenamide fumarate (TAF)) for older children/adolescents [4–6]. In high income countries (HIC) where paediatric FDCs including TAF are available, these are included in preferred recommendations from 14 kg [4,5]. WHO guidelines include TAF as an alternative first-line NRTI option [6] but it is not yet available in LMIC. Backbone recommendations for those experiencing VF vary according to factors including setting, formulation availability, previous NRTI exposure and access to genotypic resistance testing [4–6].

VF on DTG remains infrequent and genotypic resistance to INSTIs is rare, especially in the setting of first-line failure [7,19–22]. The number of children requiring options following first-line INSTI- based ART will inevitably increase and effective options for use in this context will be essential. There is currently no high-quality evidence to inform treatment options following VF on INSTI-based therapy in children. This review will summarise recently published information on paediatric INSTI use and resistance, current and future alternative ART options and highlights important research areas requiring prioritisation. 

**Box 1:**
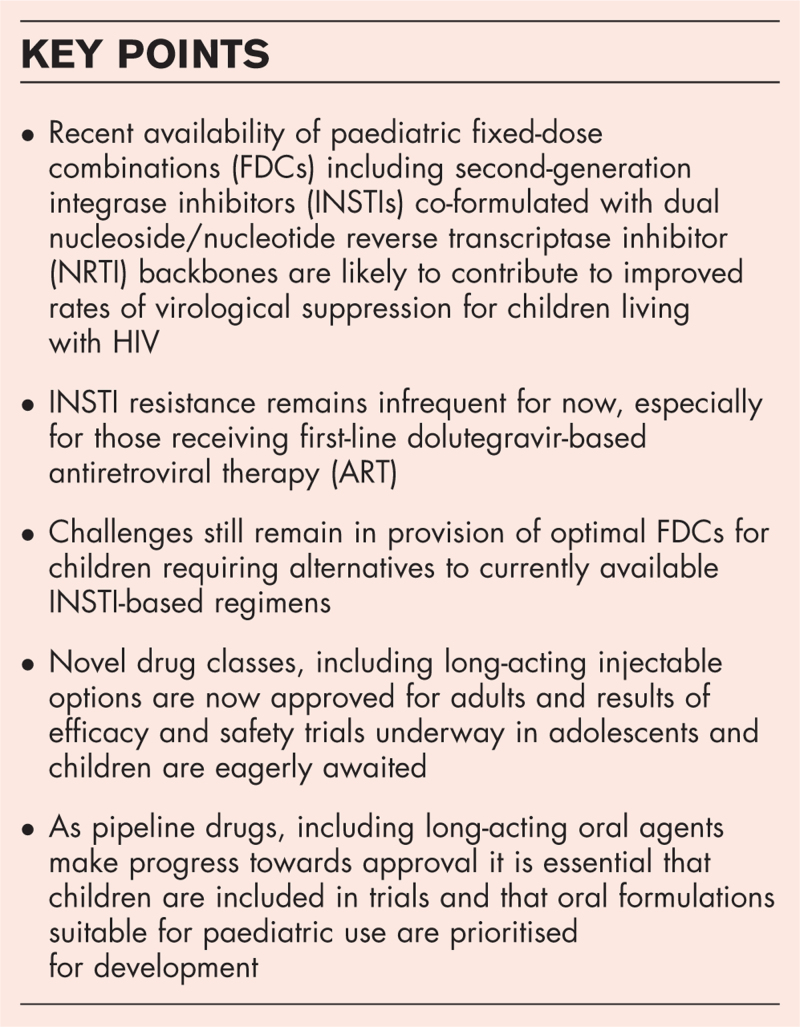
no caption available

## HOW TO ACHIEVE BETTER VIROLOGICAL SUPPRESSION IN CHILDREN

Consistent ART adherence is fundamental to avoiding VF. In addition to nonpharmacological interventions, adherence can be enhanced by providing FDCs with a high genetic barrier to resistance, palatable/convenient formulations, low pill burden, simple storage requirements, improved tolerability and simple weight-based once-daily dosing. These attributes have recently been achieved for children in HIC with approvals in place for dispersible FDC of DTG/ABC/3TC (pALD) from 6 kg [23] and paediatric bictegravir (BIC)/TAF/FTC down to 2 years/14 kg [24,25]. Evaluation of BIC/TAF/FTC tablet for oral suspension in children <14 kg is also ongoing (NCT02881320).

In LMIC, plans for rollout of generic dispersible pALD in country programmes are underway, transitioning from separate dispersible DTG (or alternative anchor drugs) and ABC/3TC coformulations. The youngest infants will still require separate dispersible DTG and ABC/3TC [26] although recent modelling data suggests that extending the use of pALD down to 4 weeks/3 kg may be possible [14].

DTG is virologically superior to ritonavir-boosted lopinavir (LPV/r) or atazanavir (ATV/r) [18] and TAF is superior to zidovudine (ZDV) or abacavir (ABC) for children requiring second-line ART after first-line NNRTI-based ART failure [27]. Development of a DTG/TAF/FTC formulation for children aged ≥4 weeks, weighing ≥3 to <25 kg is underway (UNIVERSAL-1 NCT05993767), aligning with Paediatric Drug Optimisation (PADO)-set priorities for antiretroviral drug development [28]. However, stand-alone paediatric TAF/XTC FDCs for pairing with non-INSTI anchor drugs will remain important for some treatment-experienced children following DTG-based treatment failure or ART-naïve children unable to tolerate DTG. Despite being prioritised by PADO [28], they are not yet in development by generic companies. A phase 2/3 trial of paediatric TAF/FTC with boosted PIs (cobicistat-boosted darunavir (DRV/c) ≥3 years/15 kg and atazanavir (ATV/c) ≥3 months/5 kg) is ongoing (NCT02016924) [29].

Adult guidelines recommend TDF or TAF (TXF)/XTC as preferred dual-NRTI backbone for first-line [4,6], but there is little data for TAF as an alternative to ABC for first-line treatment in children [30^▪^,^31^]. If paediatric TAF-containing FDCs become more widely available, their position in relation to ABC-based backbones for first-line treatment for young children will require consideration.

Dual therapy with DTG/3TC is now included as a first-line preferred option in HIC adult guidelines. A multinational randomised trial of DTG/3TC for suppressed-switch in children ≥2 years, including a novel dispersible FDC, is underway (NCT04337450) [32] and will provide evidence on efficacy, safety and PK, including higher than currently recommended 3TC dose within the adult FDC for children 20 to ≤25 kg. Adult data does not indicate significant additional risk of VF or resistance with DTG/3TC compared to DTG/dual-NRTI regimens and it is hoped that this will also be the case in children. There are no studies currently underway of DTG/3TC for ART-naïve children. It will be important to establish its effectiveness in this age range, especially the youngest children who frequently have high HIV viral loads and take longer to achieve virological suppression and for adolescents who may face additional adherence challenges. Guidelines are unlikely to recommend DTG/3TC first-line in children and adolescents without such additional data. Despite inclusion of DTG/3TC as an option for treatment-naive and experienced adults in HIC guidelines, its place (and that of other dual therapy options) in the public health approach provided by WHO guidelines remains to be determined [33].

Although paediatric dispersible tablets improve options for children, the importance of formulations for those who can swallow tablets, but for whom adult dosing or large adult tablets are not yet appropriate should not be forgotten. Options for children as they grow and transition from dispersible, to paediatric tablets prior to adult-dose formulations remain limited. For example, a 7-year old child on first-line ART (without access to BIC-based FDC) weighing 20–25 kg currently requires ≥4 tablets/day (e.g. DTG 50 mg + 3ABC/3TC 120 mg/60 mg FDC [26] or 1½ ABC 300 mg/1½ 3TC 150 mg once daily [34]. This should be considered as novel agents are developed for use across all age/weight bands.

## EMERGING INTEGRASE RESISTANCE

Resistance associated mutations (RAMs) to second generation INSTIs following first-line VF remain rare. Across all phase 3 first-line trials of DTG in either adults or children published to date, only one case of INSTI resistance has been reported in the VESTED trial. Small numbers of cases have also been reported in observational studies [7,19–22,35,36] (White E, Kityo C, Spyer MI, *et al.* ODYSSEY unpublished data). In trials of DTG with dual-NRTI in second-line, INSTI resistance rates of up to 3.8% have been observed [22] (White E, Kityo C, Spyer MI, *et al.* ODYSSEY unpublished data, Kityo C, Szubert AJ, Mujuru HA *et al.* CHAPAS-4 unpublished data), consistent with reports from real-world settings [20]. An observational study from Malawi demonstrated a DTG RAM prevalence of 13.5% (18/133) in a cohort of children with confirmed VF on a DTG-based regimen despite intensive adherence counselling [37]. WHO reports a 3.9–19.6% prevalence of INSTI resistance in surveys of adults with VF while receiving DTG-based ART and one case of INSTI resistance in an ART-naive infant (detected at time of HIV diagnosis at 14 months of age), whose mother was receiving DTG [19,38]. An additional case of pre-ART INSTI resistance detected at time of HIV diagnosis at 18 months of age has also recently been reported [39]. While this indicates INSTI resistance is emerging, it remains that the majority of children viraemic on DTG-based ART do not develop DTG resistance. Only limited INSTI resistance surveillance data including children is currently available and collection of unbiased survey data to estimate true prevalence will be essential [19].

Factors associated with INSTI resistance while receiving second-generation INSTIs include previous exposure to or failure on first-generation INSTI [20], the presence of NRTI resistance [40,41], suboptimal adherence [20,42] and the use of ZDV-containing NRTI backbone [22,43] (White E, Kityo C, Spyer MI, *et al.* ODYSSEY unpublished data). There is some evidence that children may be at higher risk than adults [22]. Dolutegravir RAMs appear to follow four nonoverlapping pathways; R263K, G118R, N155H and Q148H/R/K, with G118R demonstrating the greatest reduction in DTG susceptibility, and R263K and G118R most commonly reported [21,40]. Clinical risk factors associated with each pathway, including the effect of HIV subtype have yet to be elucidated [21]. Similar resistance patterns have been noted in antiretroviral experienced adolescents and children and greater accumulation of RAMs is associated with reduced DTG susceptibility [44].

The role of resistance testing in first-line VF, especially in LMIC, continues to be debated [45]. The recently presented GIVE MOVE trial in children and adolescents with viraemia in Tanzania and Lesotho, demonstrated resistance test-informed management did not improve viral suppression [46]. This aligns with findings from trials of second-line ART in adults [43,47–49] suggesting that implementing resistance testing in LMIC within current algorithms may have limited impact on virological outcomes.

However, 5 (18%) children with VF on second-line DTG therapy developed INSTI RAMs in ODYSSEY and those who developed resistance were unlikely to re-suppress while continuing on DTG-based regimens (White E, Kityo C, Spyer MI, *et al.* ODYSSEY unpublished data), highlighting the potential importance of timely DTG resistance identification in certain circumstances. Novel management algorithms should be developed focussing on identifying those at highest risk of INSTI resistance (e.g. children with persistent VF on second or subsequent-line DTG) and potentially utilising point-of-care (POC) or near-POC tests for targeted INSTI resistance evaluation [50]. For those continuing DTG, innovative methods for supporting adherence, including life-stage based discussions may improve treatment outcomes if integrated in clinical practice [51^▪^].

## WHAT ANCHOR DRUG SHOULD BE USED IF VIROLOGICAL FAILURE OCCURS ON INTEGRASE INHIBITOR-BASED ART?

If VF occurs during INSTI-based ART (± INSTI resistance), there is minimal data to guide subsequent options. If resistance testing is available and no INSTI resistance has been demonstrated, INSTI can be continued while supporting/optimising adherence. If significant INSTI resistance is demonstrated, alternative anchor drugs are required. The CHAPAS-4 trial provided robust evidence for ritonavir-boosted protease inhibitors (bPI) in second-line for children experiencing VF on first-line NNRTI-based ART, with a trend for ritonavir-boosted DRV (DRV/r) being virologically superior to ATV/r and LPV/r. Furthermore, unfavourable growth and toxicity were observed for LPV/r compared to DRV/r or ATV/r [18]. This data can be potentially extrapolated to children experiencing first-line INSTI-based VF as they are likely to have similar NRTI resistance and unlikely to have protease resistance. Given paediatric FDCs of boosted DRV and ATV are not widely available and DRV is only licensed from 3 years due to potential toxicity observed in animal studies, children under 3 years and young children unable to take DTG due to treatment failure or intolerance are currently limited to LPV/r. Additional limitations with LPV/r include twice-daily dosing, inconvenient nonpalatable liquid formulations, poor tolerability and challenging tuberculosis drug interactions. Stock-outs of paediatric LPV/r are also common [52]. Widespread consistent availability of paediatric bPI alternatives to LPV/r is an urgent unmet need. Co-formulated paediatric DRV/r has been prioritised by PADO [28]; UNIVERSAL-2 (NCT06139796) is investigating a novel paediatric FDC of DRV/r and pharmacokinetic modelling and simulation data from the study has recently been submitted to the U.S. Food and Drug Administration (FDA) for review [53]. A paediatric formulation of DRV/c/FTC/TAF 675/150/200/10 mg, suitable for children weighing 30 kg to <40 kg with current dosing recommendations is also in development [54].

In the context of extensive INSTI, NNRTI and NRTI resistance, recommendations (based predominately on expert opinion) often include twice-daily DTG and boosted DRV with 2NRTI. Achieving this combination in children is challenging and may necessitate off-label use of modified adult formulations [36]. Managing tuberculosis co-infection adds even more layers of complexity.

## WHAT NUCLEOTIDE/NUCLEOSIDE REVERSE TRANSCRIPTASE INHIBITOR BACKBONE SHOULD BE USED FOLLOWING VIROLOGICAL FAILURE?

For second-line ART, current guidelines suggest switching NRTI backbone (ABC to ZDV or TDF) or selecting NRTIs based on resistance testing [4–6]. Emerging evidence from adult and paediatric studies supports the use of TXF over ZDV for better treatment outcomes [27,43]. However, children in LMIC under 30 kg lack access to TXF formulations due to renal and bone toxicity concerns of TDF and unavailability of TAF. Consequently, the recommended NRTI backbone for these children post-ABC failure remains twice-daily ZDV. Uncertainty remains regarding continuation of ABC when transitioning to DTG-based second-line. While ABC may be less potent than TDF [55], multiple mutations are required for significant phenotypic resistance [56]. Once-daily dosing of ABC and better safety profile offer additional advantages over ZDV for children [57]. In the ODYSSEY trial, children on ABC, even with high-level ABC resistance, had lower VF rates than those on ZDV in the DTG arm (White E, Kityo C, Spyer MI, *et al.* ODYSSEY unpublished data). The BIPAI and IeDEA paediatric cohorts also reported that children retaining ABC while transitioning to DTG had high virological suppression rates [15^▪^,^58^]. Observational data confirming ABC safety and effectiveness in young infants is also now available [59]. Overall, recent evidence suggest ZDV is suboptimal for DTG-based second line, warranting guideline revision. The lack of availability of TAF FDCs, particularly in LMIC remains an unmet need to expand treatment options in children with VF. Until these formulations are available, the option of remaining on ABC rather than switching to ZDV requires further consideration.

## FUTURE OPTIONS

Novel combinations and agents for use in both treatment naïve and experienced adults are now available or in late-stage development. Long-acting oral or injectable formulations show great promise for a variety of indications. Combination long-acting injectable cabotegravir (CAB)/rilpivirine (RPV) is now licensed by the FDA (>12 years) [60] and EMA (adults only) [61] for use in suppressed switch with extensive evidence for efficacy available in HIC and LMIC [62^▪^,^63^^▪▪^]. CAB/RPV also has potential as a treatment for people with adherence difficulties [64,65,66^▪^]. Safety, pharmacokinetics, acceptability and tolerability data from phase 1/2 trials in adolescents is now available [67^▪^,^68^] and a phase 3 randomised trial in Africa including 12–19 year-olds has recently completed recruitment, comparing switch to long-acting injectable CAB/RPV with continuing suppressive DTG/TDF/3TC (NCT05154747). A phase 1/2 trial of long-acting injectable CAB/RPV in children aged 2–12 years is also ongoing (NCT05660980). Although VF is uncommon with CAB/RPV, when it occurs it can be associated with INSTI and/or NNRTI resistance. Risk factors for VF in adults include two or more of BMI>30 kg/m^2^, subtype A1/A6 and preexisting RPV RAMs [62^▪^], which should be taken into account when considering switch to CAB/RPV. It is not known whether rates and risk factors for VF will be different for children and/or adolescents.

Long-acting injectable lenacapavir (LEN) (given subcutaneously (SC) once every 6 months), a first-in-class capsid inhibitor, is licensed for adults with multidrug resistant HIV [69], efficacy having been demonstrated in the CAPELLA trial [70^▪^]. A small case series of LEN/CAB ± RPV for adults with adherence challenges has recently been reported with a high rate of virological suppression [71]. There are no data relating to use in children. Studies of daily oral and 6-monthly SC in children are planned [72] (Gilead, personal communication). For currently licensed injectable agents, it is hoped that the label can be broadened to include additional indications, especially for those who face significant challenges with adherence to oral medication.

Islatravir (ISL), a first-in-class nonnucleoside reverse transcriptase translocation inhibitor (NRTTI), is being studied as part of a once weekly oral combination (with oral LEN) in adults [72] (NCT05052996). It is unfortunate that no studies of this combination are yet underway in adolescents or children as this has potential to both simplify adherence and provide options for those with resistance. Oral fostemsavir is licensed for adults with multidrug resistant HIV [74,75], although use is not currently widespread. Studies are ongoing in children (NCT04648280). Twice daily dosing is not ideal, especially for children and/or those facing adherence challenges so its long-term place in paediatric treatment is currently uncertain. Broadly neutralising antibodies also have potential for paediatric use, however efficacy in trials completed to date is not as high as for currently licensed oral or injectable drugs [76,77]. Use in combination with other LA agents (e.g. LEN or CAB) is an area of active research [78,79] (NCT04811040, NCT03739996).

## CONCLUSION

It is hoped that universal access to paediatric formulations of DTG-based ART for all ages will bring us closer to the goals of the Global Alliance to End AIDS in Children by 2030 [80,81]. Challenges remain in providing ART following first-line failure with or without INSTI resistance, especially in the youngest children. The role of resistance testing remains to be established and surveillance for emergence of INSTI resistance will be key. Boosted PIs will remain essential for second/subsequent line and expedited development of paediatric formulations including TAF is required. Promising drugs from new classes, including long-acting options have great potential to address paediatric adherence challenges. Children must be included in trials as early as possible and their specific needs considered throughout drug development if the enduring treatment gap is to be closed [73].

## Acknowledgements

*None.*

### Financial support and sponsorship

*None.*

### Conflicts of interest

*A.B. is chair of the Penta/EACS paediatric HIV treatment guidelines working group and has received fixed-term consultancy fees from the WHO-hosted Global Accelerator for Paediatric Formulations (GAP-f). A.T. is co-chair of the WHO-led Paediatric Antiretroviral Working Group. A.T. is chief investigator (CI) for D3 trial (ISRCTN17157458) funded by Penta Foundation, ViiV healthcare and the UK Medical Research Council. A.T. and A.B. are members of the Penta-ID scientific steering committee.*

## REFERENCES AND RECOMMENDED READING

Papers of particular interest, published within the annual period of review, have been highlighted as:

▪ of special interest
▪▪ of outstanding interest
